# BMP-4 Genetic Variants and Protein Expression Are Associated with Platinum-Based Chemotherapy Response and Prognosis in NSCLC

**DOI:** 10.1155/2014/801640

**Published:** 2014-03-19

**Authors:** Sun Xian, Lang Jilu, Tian Zhennan, Zhou Yang, Hu Yang, Geng Jingshu, Fu Songbin

**Affiliations:** ^1^Department of Oncology, Third Affiliated Hospital of Harbin Medical University, 150 Ha Ping Road, Nangang District, Harbin, Heilongjiang Province 150086, China; ^2^Department of Cardiac Surgery, Second Affiliated Hospital of Harbin Medical University, 246 Xue Fu Road, Nangang District, Harbin, Heilongjiang Province 150086, China; ^3^Department of Pathology, The Third Affiliated Hospital of Harbin Medical University, 246 Haping Road, Nangang District, Harbin, Heilongjiang Province 150081, China; ^4^Laboratory of Medical Genetics, Harbin Medical University, 194 Xue Fu Road, Nangang District, Harbin, Heilongjiang Province 150086, China

## Abstract

To explore the role of genetic polymorphisms of bone morphogenic proteins 4 (BMP-4) in the response to platinum-based chemotherapy and the clinical outcome in patients with advanced nonsmall cell lung cancer (NSCLC), 938 patients with stage III (A+B) or IV NSCLC were enrolled in this study. We found that the variant genotypes of 6007C > T polymorphisms significantly associated with the chemotherapy response. The 6007CC genotype carriers had a higher chance to be responder to chemotherapy (adjusted odd ratio = 2.77; 95% CI: 1.83–4.18; adjusted < 0.001). The 6007C > T polymorphisms and BMP-4 expression also affect the prognosis of NSCLC. Patients with high BMP-4 expression had a significantly higher chance to be resistant to chemotherapy than those with low BMP-4 expression (OR = 2.81; 95% CI: 1.23–6.44; *P* = 0.01). The hazard ratio (HR) for 6007TT was 2.37 times higher than 6007CC (*P* = 0.003). In summary, the 6007C > T polymorphism of BMP-4 gene and BMP-4 tissue expression may be used as potential predictor for the chemotherapy response and prognosis of advanced NSCLC.

## 1. Introduction

Nonsmall cell lung cancer (NSCLC) accounts for approximately 80% of primary lung cancers. Many NSCLC patients diagnosed at the advanced stage (stage III or IV); thus the prognosis of NSCLC is very poor [1]. The early detection and intervention is very important to improve the clinical outcome of NSCLC.

Platinum-based chemotherapy is the standard first-line chemotherapy for advanced NSCLC; however, drug resistance is a major issue influencing the clinical outcome of patients [2, 3]. About one-third of NSCLC patients achieve remission from standard first-line chemotherapy, another one-third stable disease, and the remaining one-third progressive disease [4].

Many clinical factors, such as age, cancer stage, differentiation status, and cancer metastasis, have been considered as major determinations for prognosis of NSCLC [5]. However, variability in prognosis has still been observed in patients with similar clinical features. The host inherited factors, for example, genetic background, may have an important role in determining the treatment outcome for NSCLC [6–9]. Recent studies revealed that certain genetic polymorphisms are associated with the treatment response of platinum-based chemotherapy in advanced NSCLC [10–14].

As a member of bone morphogenic proteins (BMP) superfamily, BMP-4 is a multifunctional growth factor belonging to the TGF-*β* super family which is known to play an important role in regulating osteoblast differentiation and bone formation [15–18]. Recently, a study showed that BMP-4 is highly expressed in cisplatin-resistant gastric cancer (GC) cell lines. Targeted genetic inhibition of BMP-4 caused significant sensitization of GC cells to cisplatin. This study suggests that BMP4 epigenetic and expression status may represent promising biomarkers for cisplatin resistance in cancer cells [19].

Genetic variants in BMP-4, in the form of single nucleotide polymorphisms (SNPs), may result in a qualitative or quantitative change in the local production of BMP-4 or in its effectiveness via its cognate receptor [20]. Based on the role of BMP-4 in drug resistance in GC cell lines, we assumed that there might be an association between BMP4 genetic variants and chemotherapy response in cancer patients. In the present study, we enrolled NSCLC patients undergoing platinum-based chemotherapy to explore the association between the BMP4 genetic variants and chemotherapy response as well as the prognosis of NSCLC.

## 2. Patients and Methods

### 2.1. Patient Enrollment

A total of 938 patients with stage III to IV NSCLC were consecutively recruited between May, 2006, and July, 2011. All the patients were enrolled according to the criteria described elsewhere [21]. Patients were excluded if they had a prior history of malignancy; previous chemotherapy, radiotherapy or surgery; active congestive heart failure, cardiac arrhythmia, or recent (<3 mo before the date of treatment) myocardial infarction; any severe mental disorder; infectious disease needing immunotherapy. The study was carried out with the approval of the Ethical Review Committee of the hospital. A written informed consent was provided by each patient. Data on demographic and clinical characteristics (including age, sex, smoking status, and tumor histology) were obtained from clinical medical records with review by the oncologists.

### 2.2. Platinum-Based Chemotherapy

The chemotherapeutic regimens were described previously [21]. All chemotherapy drugs were administered intravenously, and all treatments were for two to six cycles. Patient responses to treatment were determined after four cycles by the WHO criteria, which classify the response into four categories: complete response (CR), partial response (PR), stable disease (SD), and progressive disease (PD). CR was defined as complete disappearance of all measurable lesions. PR required at least 50% reduction in measurable lesions. Patients with SD had less than a 50% decrease or no more than a 25% increase in the size of measurable lesions. PD was assigned to patients when measurable lesions increased by more than 25% or new lesions appeared. For data analysis, CR and PR were combined as responders, and SD and PD were grouped as nonresponders [22, 23].

### 2.3. Genotyping and SNPs of BMP-4 Gene

DNA was extracted from peripheral whole blood using a Qiagen DNA Isolation Kit (Qiagen, Valencia, CA, USA). The specific product of the BMP-4 gene was amplified by polymerase chain reaction (PCR) with primers 5′-biotion-TGAAGGCAAGATGTCTGA-CACA-3′ (forward) and 5′-CCTTCCTGCATTTCTATC-CTA-3′ (reverse) for −5826G > A (rs1957860) and 5′-ATTGCCCAACCCTGAGCTATC-3′ (forward) and 5′-biotin-TGGGGGCTTCATAACCTC-3′ (reverse) for 6007C > T (rs17563). Reactions were performed in a total volume of 20 *μ*L. The thermocycling procedure consisted of initial denaturation at 95°C for 3 minutes, 35 cycles of denaturation at 94°C for 30 seconds, annealing at 60°C for 40 seconds, extension at 72°C for 1 minute, and a final extension at 72°C for 10 minutes. The PCR products were analyzed by electrophoresis on 1% agarose gel.

### 2.4. BMP-4 Immunohistological Staining

Tumor samples were obtained from the biopsy before the start of chemotherapy. Anti-BMP-4 polyclonal antibody was used in the immunohistological staining at a 1 : 250 dilution. Sample scoring was performed by semiquantitative microscopic analysis, considering the number of stained cells and signal intensity. Two spots were evaluated for each sample and a mean score was calculated. Considering the percentage of BMP-4 immune-positive tumor cells, a score of 1 was given when ≤10% of cells were positive; 2 when 10–50% of cells were positive, and 3 when ≥50% of cells were positive. Signal intensity was scored as negative (0), weak (1), moderate (2), and strong (3). Both scores were multiplied [24] and the resulting score was used to categorize BMP-4 expression as low (0–6) and high (>6) expressions.

### 2.5. Western Blot Analysis

Tumor samples were lysed for western blot assay to determine BMP-4 expression. After immunoblot analysis, membranes were immunoblotted with BMP-4 antibody and GAPDH (both 1 : 1000, Santa Cruz Biotechnology, Santa Cruz, CA). Membranes were then washed and incubated with a secondary antibody coupled to horseradish peroxidase. To evaluate the amount of protein expression, the Raytest TINA software (http://www.raytest.de/service/raytest_catalog.html) was used for western blotting to calculate the densitometric analysis as described previously [25]. The differences in the relative expressions among different genotypes were compared by using ANOVA analyses.

### 2.6. Statistical Analysis

Data on quantitative characteristics are expressed as means ± SD. Differences in demographic characteristics and vascular risk factors between patients and controls were compared by using Student's *t*-test or ANOVA for continuous variables and the *χ* ^2^-test for all categorical variables. To estimate the deviation of frequency of gene alleles in tested population, we performed the Hardy-Weinberg equilibrium using *χ* ^2^ tests. Genotypes and allele frequencies were compared by *χ* ^2^ or fisher analysis or Fisher's exact test. Overall survival (OS) and progression free survival (PFS) were the end points in this study. OS was calculated from the date of chemotherapy to the date of last followup or death from any cause. PFS was defined as the interval between the date of chemotherapy and the date of confirmed relapse. Survival was analyzed using the Kaplan-Meier method. The log-rank test was used to analyze survival differences. Multivariate logistic regression analysis was used to determine the influence of BMP-4 polymorphisms on chemotherapy response, controlling potential confounding conventional risk factors. A forward stepwise (Likelihood Ratio) procedure was used for multivariable analysis. Multivariate Cox proportional hazards regression models were performed to estimate the hazard ratios (HR) for overall survival and their 95% CIs. Data were analyzed with the SAS 9.2 package (SAS Inc., NC, USA). The results were considered statistically significant at *P* < 0.05.

## 3. Results

### 3.1. Patient Characteristics and Clinical Outcomes

Of all the participants, 364 were assigned into responder group (CR + PR) and 574 were assigned into nonresponder group (SD + PD).  Table 1 shows demographic and clinical characteristics between 2 groups. Nonresponders were older, who had higher prevalence of smokers, stage IV, and poor differentiation patients than responders (all *P* < 0.05, Table 1). There were no significant differences in sex, histology type, and chemotherapy regimens between responders and nonresponders (all *P* > 0.05).

Genotype frequencies of BMP-4 polymorphisms in chemotherapy responder and nonresponders were found to be in Hardy–Weinberg equilibrium (all *P* > 0.05). The genotype and the allele frequencies of −5826G > A were not significantly different between chemotherapy responder and nonresponders. However, the genotypes and allele frequency of 6007C > T were significantly different between the responders and nonresponders. Responders had a markedly higher CC genotype than responders (34% versus 21%; overall *P* < 0.0012, Table 2). With TT as reference, multivariate logistic regression analysis showed that the CC genotype carriers had a higher chance to be responder to chemotherapy (adjusted OR = 2.77, 95% CI: 1.83–4.18, adjusted *P* < 0.001) with adjustment for age, sex, smoke status, histology, stage, and chemotherapy agents. The C allele carriage represented a higher possibility of being responders to chemotherapy after adjustment with the above-mentioned clinical variables (adjusted OR = 1.71, 95% CI: 1.39–2.11, *P* < 0.001) compared with T allele carriage. However, multivariate logistic regression analysis did not reveal any association between −5826G > A polymorphisms and chemotherapy response in these patients (all *P* > 0.05, Table 2).

**Figure 1:**
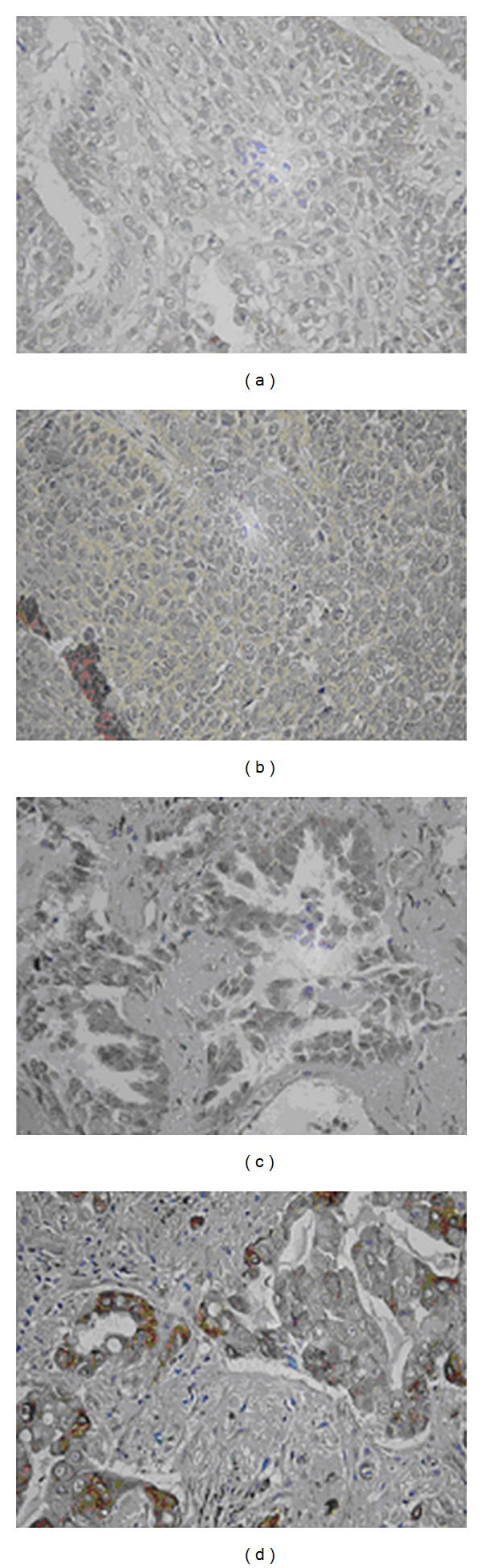
Representative figures of BMP-4 staining from different NSCLC patients. (a) BMP-4 low expression in squamous cell carcinoma. (b) BMP-4 high expression in squamous cell carcinoma. (c) BMP-4 low expression in adenocarcinoma. (d) BMP-4 high expression in adenocarcinoma.

The BMP-4 expression levels in biopsy tumor samples were determined by immunohistological staining. 93 samples were obtained from studied participants, of which 36 were responders to chemotherapy, while 57 were nonresponders. The BMP-4 high expressions were observed in 45 of nonresponders (78.9%) but only in 12 of responders (33.3%).  Table 3 shows the association between the BMP-4 expression and chemotherapy response status. Patients with high BMP-4 expression had a significantly higher chance to be resistant to chemotherapy than those with low BMP-4 expression (OR = 2.81, 95%CI: 1.23–6.44, *P* = 0.01) after adjustment with age, sex, smoke status, histology, stage, and chemotherapy agents. Representative figures of BMP-4 staining were shown in Figure 1.

**Figure 2:**
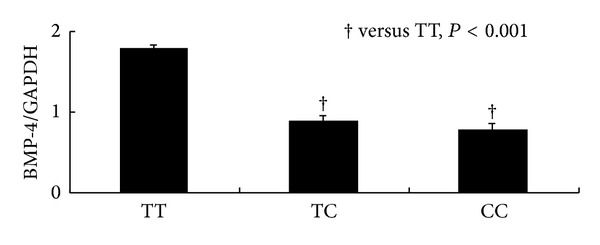
Quantitative analyses of western blot bands of BMP-4 in 6007C > T genotype carriers.

The association between the BMP-4 genotype and the BMP-4 protein expression in tumor samples was analyzed.  Table 4 shows that the TT carriers of 6007C > T had a significantly higher rate of high BMP-4 expressions, while the CC carriers had lower chance of high BMP-4 expressions (47% versus 17%, *P* = 0.025). Quantitative analyses of western blot bands also showed that the BMP-4 expression was highest in TT carriers compared to CC and CT carriers (*P* < 0.001, Figure 2).

**Figure 3:**
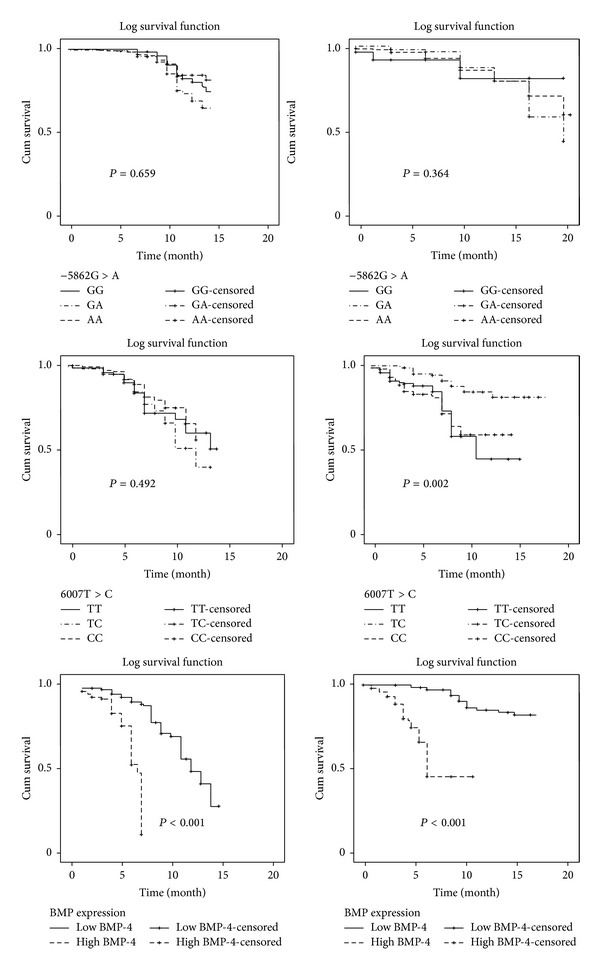
The Kaplan-Meier curves for BMP-4 gene polymorphism and expression for PFS and OS.

The associations between the clinical variables and PFS as well as OS were studied by log-rank test (Table 5). For PFS, there were no significant differences in PFS with respect to BMP-4 genotypes at −5826 and 6007 loci (both *P* > 0.05 by log-rank analyses). For OS study, 6007C > T polymorphisms showed statistically significant associations with OS by log-rank test. The median survival time for patients with CC was significantly longer than that in the CT and TT genotype carriers after adjustment for age, sex, performance status, cancer stage, and treatment regimens (*P* = 0.002, Table 5). The BMP-4 expression determined both PFS and OS. The patients with high BMP-4 expressions had a significantly shorter PFS and OS compared with those with low BMP-4 expression levels (both *P* < 0.001). The Kaplan-Meier survives curve are shown in Figure 3.

Multivariate Cox proportional hazards regression models were performed to estimate the hazard ratios (HR) for overall survival and their 95% CIs, with adjustment for age, sex, smoke status, histology, stage, and chemotherapy status (Table 6). The HR for 6007TT was 2.37 (95% CI: 1.89–3.98 compared with CC carriers, *P* = 0.003). The SNPs of −5826G > A did not show this trend in the evaluation of their role in determining the prognosis of NSCLC subjects (*P* = 0.072). The HR of BMP-4 expression was 3.87 (*P* < 0.001).

## 4. Discussion

In this study we evaluated whether BMP-4 polymorphisms influence the treatment response and clinical outcomes of Chinese NSCLC patients treated with platinum-based chemotherapy. We found the following. (1) The variant genotypes of 6007C > T polymorphisms significantly associated with the chemotherapy response. The CC genotype carriers had a markedly higher chance to be responders to chemotherapy. (2) The polymorphism at 6007C > T affects the prognosis of NSCLC as the OS period was significantly shorter in TT carriers than CC carriers. (3) The BMP-4 expressions were significantly associated with the chemotherapy response. Patients with high BMP-4 expression were more likely to be resistant to chemotherapy than those with low BMP-4 expression. (4) The 6007C > T polymorphisms affect the BMP-4 protein expression in tumor samples. The TT carriers of 6007C > T had a significantly higher rate of high BMP-4 expressions, while the CC carriers had lower chance of high BMP-4 expressions.

Although many mutations within BMP-4 leading to various phenotypes have been reported [26], the single nucleotide polymorphism of 6007C > T (rs17563) of BMP-4 is the only identified polymorphism in the coding region [27]. The 6007C > T polymorphism results in an amino acid change from valine to alanine at residue 152 (p.Val152Ala) and thus affects the BMP-4 gene expression. The T-allele was predicted to change mRNA structure and the BMP-4 mRNA levels were significantly higher in T-allele carriers compared with C-allele carriers; even the BMP4 protein plasma levels were higher among T-allele carries, but without reaching the statistical significance [28]. In this study, we found that the TT carriers of 6007C > T had a significantly higher rate for having high BMP-4 expressions, while the TT carriers had markedly higher percentage of high BMP-4 expressions, consistent with the result of above-mentioned study [28].

Recently, BMP-4 was shown to affect the response of cancer cells to the antitumor reagents. A previous study showed that the BMP-4 induces differentiation of cancer stem cells in colorectal tumor and increases their response to chemotherapy in mice, suggesting that BMP-4 might be developed as a therapeutic agent against cancer stem cells in advanced colorectal tumors [29]. BMP-4 epigenetic and expression status may represent promising biomarkers for GC cisplatin resistance [19, 30]. An epigenomic analysis identified BMP-4 as an epigenetically regulated gene highly expressed in cisplatin-resistant lines. Functional assays confirmed that BMP4 is necessary and sufficient for the expression of several prooncogenic traits [19]. Administration of BMP-4 to immunocompromised mice with tumors that arose from CRC-SCs increased the antitumor effects of 5-fluorouracil and oxaliplatin [29]. These studies prompted us to testify whether the BMP-4 genetic variants affect the chemotherapy response in NSCLC cancer patients. We found a significant association between the BMP-4 polymorphisms at 6007C > T and the response to platinum-based chemotherapy, although the mechanism remains to be explored.

The prognostic role of BMP-4 in cancer prognosis has been previously reported. In serous ovarian carcinoma, strong expression of BMP-4 before chemotherapy was an independent prognostic factor of longer progression-free time and overall survival, but it was not associated with neovascularization [31]. The correlation of BMP-4 expression with clinical aggressiveness and prognosis in hepatocellular carcinoma (HCC) has also been reported. The expression of BMP-4 in HCC was associated with number of tumor nodules, Edmondson grade, TNM stage, and vascular invasion. BMP4 protein expression was found to be an independent factor for predicting both overall and disease-free survival of HCC [32]. In this study we found that the BMP-4 protein expression was closely related to both PFS and OS in NSCLC patients. Collectively, these studies suggest that BMP4 expressed in tumor tissues may act as a novel marker for predicting the prognosis of cancer patients, including patients with NSCLC.

Several limitations in this study need to be addressed. This study was a single-center cohort investigation on a relatively small scale. A validation study, either in an independent cohort of patients or functional assay, is lacking in this study. Secondly, we did not detect the BMP4 expression in either serum or cancer tissue of NSCLC subjects. The detection of BMP4 protein will help to clarify the effect of the genetic variants of BMP4 gene on chemotherapy response.

## Figures and Tables

**Table 1:** Patient characteristics between chemotherapy responder and nonresponders.

Characteristics	Responders	%	Nonresponders	%	*P*
Age (years)	55.6 ± 6.6		60.7 ± 5.2		0.041
Gender					
Male	207	56.9%	328	57.1%	0.494
Female	157	43.1%	246	42.9%	
Smoke status					
Nonsmokers	84	23.1%	89	15.5%	<0.001
Smoker	280	76.9%	487	84.5%	
Histology type					
Squamous cell carcinoma	208	57.1%	321	55.9%	0.383
Adenocarcinoma	156	42.9%	253	44.1%	
Stage					
IIIA	156	42.9%	187	32.6%	0.005
IIIB	119	32.7%	211	36.8%	
IV	89	24.5%	176	30.7%	
Differentiation					
Well	134	36.8%	165	28.7%	0.019
Moderate	129	35.4%	210	36.6%	
Poor	101	27.7%	199	34.7%	
Chemotherapy regimens					
DDP/CBP + TAX/TXT/DOC	156	42.9%	233	40.6%	0.168
DDP/CBP + GEM	121	33.2%	224	39.0%	
DDP/CBP + NVB	87	23.9%	117	20.4%	

NS: not significance; DDP: cisplatin; CBP: carboplatin; TAX: taxol/paclitaxel; TXT: taxetere; DOC: docetaxel; GEM: gemcitabine; NVB: vinorelbine.

**Table 2:** The genotype frequencies for BMP-4 polymorphisms between responders and nonresponders.

	Responders		Nonresponders		Adjusted OR^†^, 95% CI	Adjusted P^†^
	*N* = 364	%	*N* = 562	%		
*−*5826G > A						
GG	112	31%	184	32%	1	
GA	171	47%	283	49%	0.99, 0.73–1.34	0.96
AA	81	22%	107	19%	1.24, 0.86–1.80	0.25
G	395	54%	651	57%	1	
A	333	46%	497	43%	1.12, 0.92–1.33	0.3
6007C > T						
TT	70	19%	179	31%	1	
CT	167	46%	278	48%	1.34, 0.98–2.11	0.051
CC	127	35%	117	20%	2.78, 1.91–4.03	<0.001
T	307	42%	636	55%	1	
C	421	58%	512	45%	1.72, 1.41–2.05	<0.001

^†^Adjusted with age, sex, smoke status, histology, stage, and chemotherapy agents.

**Table 3:** The association between the BMP-4 expression and chemotherapy response status.

BMP-4 expression	Nonresponders		Responders		OR^†^	95% CI^†^		*P* ^†^
Low	32	41.56%	24	66.67%	1.00			
High	45	58.44%	12	33.33%	2.81	1.23	6.44	0.01

^†^Adjusted with age, sex, smoke status, histology, stage, and chemotherapy agents.

**Table 4:** The association between the BMP-4 genotype and the BMP-4 protein expression in tumor samples was analyzed.

Genotype	High BMP-4 expression *N* = 36	%	Low BMP-4 expression *N* = 57	%	*P* ^†^
−5826G > A					
GG	11	31%	19	33%	0.961
GA	15	42%	23	40%	
AA	10	28%	15	26%	
6007C > T					
TT	17	47%	13	23%	0.025
CT	13	36%	21	37%	
CC	6	17%	23	40%	

^†^Adjusted with age, sex, smoke status, histology, stage, and chemotherapy agents.

**Table 5:** The associations between BMP-4 polymorphisms and PFS and OS of NSCLC patients undergoing chemotherapy.

BMP-4 polymorphisms	Median PFS, mo (95% CI)	Log-rank *P* ^†^	Median OS, mo (95% CI)	Log-rank P^†^
−5826G > A				
GG	8.4 (4.5–10.2)	0.659	17.5 (7.6–22.4)	0.364
GA	7.9 (5.0–9.1)		18.1 (7.7–20.8)	
AA	8.2 (4.9–7.7)		17.1 (6.9–19.5)	
6007C > T				
TT	6.6 (5.5–8.2)	0.492	13.5 (5.6–18.8)	0.002
CT	7.0 (4.7–11.5)		14.7 (6.8–19.4)	
CC	8.9 (5.4–12.1)		18.4 (11.8–19.5)	
BMP-4 expression				
Low	10.0 (8.1–17.5)	<0.001	18.6 (9.9–23.2)	<0.001
High	6.2 (4.9–10.7)		9.8 (5.1–13.2)	

^†^Adjusted with age, sex, smoke status, histology, stage, and chemotherapy agents.

**Table 6:** HR for prognosis of NSCLC patients undergoing chemotherapy.

Factors	HR^†^	95% CI^†^	P^†^
6007C > T			
CC	1		
TT	2.37	1.58–3.98	0.003
BMP-4 expression			
Low	1		
High	3.87	1.37–4.25	<0.001

^†^Adjusted with age, sex, smoke status, histology, stage, and chemotherapy agents.
